# Microplastic Particles Detected in Fetal Cord Blood, Placenta, and Meconium: A Pilot Study of Nine Mother–Infant Pairs in South China

**DOI:** 10.3390/toxics12120850

**Published:** 2024-11-26

**Authors:** Minting Zhu, Xiaotian Li, Wei Lin, Dan Zeng, Pan Yang, Weigui Ni, Zhijian Chen, Bingyi Lin, Lijuan Lai, Zhongai Ouyang, Jingjie Fan

**Affiliations:** 1 School of Public Health, Southern Medical University, No. 1023-1063, Shatai South Road, Baiyun District, Guangzhou 510515, China; minti_zhu@163.com (M.Z.); m13487358979@163.com (Z.O.); 2 Department of Preventive Healthcare, Shenzhen Maternity and Child Healthcare Hospital, Southern Medical University, No. 2004 Hongli Road, Futian District, Shenzhen 518028, China; sfynwg@protonmail.com (W.N.); zj_chen07@163.com (Z.C.); linbingyi0@163.com (B.L.); lailj@mail2.sysu.edu.cn (L.L.); 3 Department of Obstetrics, Shenzhen Maternity and Child Healthcare Hospital, Southern Medical University, No. 2004 Hongli Road, Futian District, Shenzhen 518028, China; xiaotianli555@163.com; 4 Department of Healthcare, Shenzhen Maternity and Child Healthcare Hospital, Southern Medical University, No. 3012 Fuqiang Road, Futian District, Shenzhen 518028, China; linweivicle@126.com; 5 Department of Pharmacy, Shenzhen Maternity and Child Healthcare Hospital, Southern Medical University, Shenzhen 518048, China; zengdan_301@163.com; 6 School of Basic Medical Sciences, Jinan University, Guangzhou 510632, China; yangpan@jnu.edu.cn

**Keywords:** microplastics, cord blood, placenta, meconium, mother–infant pair, lifestyle

## Abstract

Microplastics (MPs) are emerging environmental pollutants. Pregnancy and infancy are sensitive windows for environmental exposure. However, few studies have investigated the presence of MPs in mother–infant pairs, or the exposure source. In this study, nine mother–infant pairs were recruited, and samples of placenta, cord blood, and meconium were collected. Information about the living environment and dietary habits were collected to determine the source of exposure during pregnancy. Micro-Raman spectroscopy was applied to identify MPs. In total, 9, 4, and 14 types of MPs were identified in the placenta, cord blood, and meconium samples, with particle counts of 34, 14, and 80, respectively. More than 80.47% of MPs detected in samples had a size of 100–400 μm. The abundance of MPs exhibited the order of meconium > placenta > cord blood (Hc = 14.959, *p* < 0.01). We found that the abundance of MPs in meconium from women who drank tea ≥ 3 times/week during pregnancy was lower than in those who drank less (*p* = 0.048). Our study presents evidence of MPs transfer via the placenta–cord blood–meconium pathway. We also found that the habit of drinking tea among pregnant women might be related to the abundance of MPs in meconium.

## 1. Introduction

Microplastics (MPs), defined as plastic particles, fibers, or films with a diameter of ≤5 mm, are a new environmental pollutant. MPs are divided into primary and secondary. Primary microplastics may be directly produced from the manufacture of micro-sized particles for household or industrial uses, while secondary MPs may be derived from the degradation of large plastic items through solar radiation, heat, and mechanical abrasion [1,2,3]. MP pollution raises concerns as plastic is ubiquitous in the living environment and could have potential adverse effects on human health [4]. A multicenter cohort study published in the *New England Journal of Medicine* has, for the first time, demonstrated that exposure to MPs has adverse effects on human health. The study found that participants with detectable MPs in their arterial samples had a 4.53-fold increased risk of developing heart disease, stroke, and all-cause mortality in subsequent follow-ups compared to those who did not [5]. Moreover, animal experiments have demonstrated that exposure to MPs can induce cardiotoxicity [6], hepatotoxicity [7,8], reproductive toxicity [9,10,11], alterations in lipid metabolism [12], and impaired gut function [13,14]. Pregnant women and fetuses, as a specific group, are extremely sensitive to environmental pollutants. Studies have confirmed that exposure to micro- and nanoplastics during pregnancy can cause maternal–fetal immune disorders [15], change in placental metabolism [16], and placental dysfunction [17]. Furthermore, it also has adverse impacts on the health of offspring, such as abnormal brain structures [18], metabolic disorders [19], and growth restrictions [20]. Therefore, it is crucial to focus on the exposure of mothers and fetuses to MPs during pregnancy.

Due to the advantages of their affordability and convenience, plastic products have become a part of human life, but they also bring a series of pollution issues. It is reported that 353 million tons of plastic waste were generated in 2019 [21]. By 2060, plastic waste is expected to triple to more than 1 billion tons [21], which poses a great threat to the human living environment, especially in rapidly developing urban regions, such as Shenzhen, China. Today, MP pollution in Shenzhen is severe [22], and the ecological risk assessment indicates a high level of risk [23]. In addition, previous studies have detected MPs in everyday items (such as toothpaste, scrubs, masks, etc.) [24,25,26]; drinking water (tap water, bottled water) [27,28,29]; food and condiments (e.g., takeout, seafood, table salt, etc.) [27,29,30,31]; and beverages (soft-packaged drinks, tea, honey, etc.) [32,33,34]. Notably, MPs can also enter the human body. A study that identified the presence of MPs in human feces raised considerable concern in 2019 [35]. Subsequently, growing evidence has shown that MPs are pervasive in the human body, with them having been detected in the lungs [36], heart [37], placenta [38,39], endometrium [40], testes [41], blood [42], sputum [43], urine [44], semen [41], amniotic fluid [38], meconium [39], and infant feces [39]. Umbilical cord blood, acting as a vital medium for the exchange of gases, the supply of nutrients, and the elimination of metabolic waste between the mother and the fetus, is crucial for maintaining normal fetal growth in utero. Nevertheless, the epidemiological evidence of MPs in human cord blood is currently scarce.

The lifestyle is associated with the exposure levels of MPs in human biological samples. A study demonstrated a moderate correlation between the intake of beverages and the abundance of MPs in feces [45]. A study found that the abundance of MPs in the endometrium from participants who drank and chewed gum was higher compared to those who did not [40]. Elsewhere, a study found that the MPs in human sputum were related to individual smoking habits [43]. Another study explored the relationship between lifestyle during pregnancy and the abundance of MPs in the placenta, discovering that water intake and the use of scrub cream or toothpaste may be potential sources of MPs in the placenta [46]. However, there is still a dearth of evidence on the relationship between the maternal lifestyle during pregnancy and biological samples’ levels of MPs.

The choice of MP detection technology plays a significant role in the identification of MPs. Different studies, sample types, and collection methods lead to different separation and analysis techniques. Nowadays, techniques such as laser direct infrared spectroscopy (lD-IR), pyrolysis gas chromatography/mass spectrometry (Pyr-GC/MS), Fourier transform infrared spectroscopy (FTIR), and Raman spectrometry are commonly used for the detection and identification of MPs in human tissues and body fluids [42,46,47,48,49,50]. Raman spectroscopy is a commonly used technique for the detection of micrometer-sized plastic fragments in complex matrices [51]. It combines with an optical microscope to focus the laser onto a small area with a high spatial resolution and minimal interference from water, making it suitable for identifying MPs larger than 1 μm.

Therefore, to investigate the exposure to MPs in mothers and infants, we used micro-Raman spectroscopy to qualitatively and quantitatively determine the characteristics of MPs in placenta, umbilical cord blood, and meconium. Also, we integrated questionnaire data on the living environment and dietary habits of pregnant women to explore the potential sources of MP contamination in the placenta, cord blood, and meconium. The results provide preliminary evidence of a relationship between MP exposure and lifestyles. They also provide the necessary evidence for subsequent environmental management, especially for plastics.

## 2. Materials and Methods

### 2.1. Study Population

In 2023, a total of 9 pairs of hospitalized mothers were recruited for this study at the Department of Obstetrics of Shenzhen Maternity and Child Healthcare Hospital, Southern Medical University, China. All volunteers were free of heart disease, chronic kidney disease, psychiatric disorders, hypertension, diabetes, gestational hypertension, and gestational diabetes. The women were asked to fill out a questionnaire to record their demographic information, characteristics of their living environment, and eating and lifestyle habits during face-to-face interviews. Informed consent was obtained from all subjects involved in our study, who all completed the questionnaire. Nine placentas, nine umbilical cord blood samples, and nine meconium samples were collected, respectively. This study was conducted in accordance with the Declaration of Helsinki, and the protocol was approved by the Medical Ethics Committee of Shenzhen Maternity and Child Healthcare Hospital (No. SFYLS[2023]028).

### 2.2. Questionnaire Survey

The questionnaire investigated newborn and pregnant women’s demographic information, characteristics of the living environment, and eating and lifestyle habits during pregnancy. Demographic information included age, body mass index (BMI) before pregnancy, education level (high school and below, bachelor’s degree, postgraduate and above), annual income per capita (<100 thousand CNY, 100–200 thousand CNY, >200 thousand CNY), occupation (governmental official, profession and technology, general office, business services, others), parity (primipara, multipara), mode of pregnancy (natural, assistance), birth outcome (preterm, full-term), mode of delivery (vaginal delivery, cesarean), sex of newborn (male, female), and newborn weight. Characteristics of the living environment included having sites of air pollution around your home or not (yes/no), distance of residence from major transportation routes (≤100 m, >100 m), and frequency of using air purifiers at home (never/seldom/often). The eating and lifestyle during pregnancy included the source of drinking water (tap water, barreled pure water), passive smoking (yes/no), drinking water in plastic cups/bottles (never/seldom/often), milk tea consumption (never/seldom/often), tea consumption (never/seldom/often), drinking beverages in plastic bottles (never/seldom/often), microwave food using plastic tableware (never/seldom/often), take-out frequency (never/seldom/often), eating seafood (fish, shellfish), drinking boxed milk (never/seldom/often), scrub cleanser (never/seldom/often), toothpaste (never/seldom/often), and daily time wearing a facemask (<3 h/day, ≥3 h/day). The questionnaire is shown in Table S1.

### 2.3. Sample Collection

Samples were collected by trained obstetricians and midwives. After delivery, umbilical cord blood was extracted by a sterile disposable syringe (JERONG, 5 mL, Weihai, China, individually packaged) [37] and then released into a glass anticoagulant tube (KERONG, 3 mL, Chengwu, China). The placentas taken from the fetal side close to the umbilical cord were deposited in a sterile stainless steel container and placed in specimen boxes. Meconium was collected by trained nurses or family members. The top portion of meconium was collected by wooden cotton swabs (12 cm, Foshan Kangzheng, Foshan, China) from the surface of the diaper and placed in a specimen box during the first 24 h after delivery. To avoid contamination, all consumables in contact with the samples during the sampling process were non-plastic products. All samples were stored at −80 °C. All samples were strictly anonymous and were shipped to a third party (Kafo, Nanjing, China) for identification.

### 2.4. Microplastic Extraction

The collected meconium and placenta samples were lyophilized by a freeze-dryer (Labconco, FreeZone, Kansas City, MO, USA). All samples including those freeze-dried and the cord blood were digested using the acid digestion method referenced by the previous studies [50,52]. Briefly, 0.1–1.0 g of sample was weighed and placed in a pre-cleaned beaker. We added 10 mL of hydrogen peroxide (30%, *v*/*v*, Guangzhou Chemistry, Guangzhou, China) for digestion at 70 °C for 3 h. Then, 2 mL of concentrated nitric acid (60%, *v*/*v*, Guangzhou Chemical, Guangzhou, China) was added, and the digestion continued for 2 h. Finally, the digestion solution was filtered using a glass fiber membrane (Jinjing, Shanghai, China) with a pore size of 1 μm. The obtained filtered membrane was collected for MP analysis.

### 2.5. Microplastic Detection

Here, a micro-Raman spectrometer (Renishaw, inVia-Reflex, Wotton-under-Edge, Gloucestershire, UK) was used to analyze and determine the physical properties and chemical composition of suspected MPs, based on previous related studies [13,50]. The specific parameters analyzed were as follows: 785 nm laser, 200–1100 nm scanning range, 1% exposure intensity, and 0.2 s exposure time. The spectra of each potential polymer were subjected to an adjusted baseline and smooth processing. The Raman spectra of the target particles were compared with an in-house library of Renishaw (WiRE 3.4), and the plastic material with a similarity rate higher than 40% in the software recommendation was used as a threshold to identify the polymer types of the MPs. The size, shape, and type of confirmed MPs were recorded.

### 2.6. Quality Assurance and Quality Control

The reagents (hydrogen peroxide, concentrated nitric acid, ultrapure water, and anhydrous ethanol) were filtered through a glass fiber membrane with a pore size of 1 μm before use. Placenta and meconium sampling tools were stainless steel products, and specimen boxes were urine cups pretreated with tinfoil, all of which were rinsed three times with anhydrous ethanol before use, and then rinsed three times with ultrapure water. There was no direct contact with plastic products during sample collection, digestion, and testing.

To exclude the influence of non-experimental factors on the results, according to the difference in sampling treatment, this study made three blank controls in the whole process of placenta-feces and umbilical cord blood collection and treatment, respectively. Few MPs were identified in the three blank controls of placenta-feces, indicating a good-quality control. Moreover, the recovery capacity of the detection method was considered. To verify the detection method in our study, we selected the three most common plastics (polyethylene (PE), polystyrene (PS), and polyvinyl chloride (PVC)), and assessed their recyclability rates. The recovery rates of the three targeted MPs (PE, PS, and PVC) ranged from 80 to 110%, which was consistent with the levels of previous studies [53].

### 2.7. Statistical Analyses

Skewed-distribution continuous variables were described by the median and interquartile range (IQR), and the categorical variables were described by number and percentage (%). The differences in the abundance (particles/gram) between different types of samples were analyzed by the Kruskal–Wallis H test. Spearman correlation analysis was conducted to examine the correlation between placenta, cord blood, meconium, and MP abundance. The Mann–Whitney U test was utilized to examine differences in the abundance (particles/gram) of MPs in placenta, cord blood, and meconium according to the living environment and the lifestyle during pregnancy. All statistical analyses were performed using SPSS 25.0. All statistical tests were two-sided, and a value of *p* < 0.05 was defined as statistically significant.

## 3. Results

### 3.1. Characteristics of the Participants

The demographic characteristics of participants are summarized in Table 1. The median BMI before pregnancy and the median age were 21.3 kg/m^2^ and 31.8 years old, respectively. All pregnant women were conceived naturally, with four of them being primiparous. The numbers of full-term and preterm infants were five and four, respectively, with a median birth weight of 2980 g.

### 3.2. Microplastics in the Placenta, Cord Blood, and Meconium

One hundred and twenty-eight MP particles were detected in the samples collectively, as shown in Table 2. MPs were detected in all placenta and meconium samples, with a total of 34 and 80 particles, respectively. MPs were detected in five out of nine cord blood samples, with a total of 14 particles. The abundance of MPs exhibited the order of meconium > placenta > cord blood (Hc = 14.959, *p* < 0.01).

In the placenta, the highest abundance of MPs detected was 9.15 particles/g, and the lowest was 1.37 particles/g. In the meconium, the highest abundance of MPs identified was 77.17 particles/g, and the lowest was 2.23 particles/g. In the cord blood, the highest abundance of MPs determined was 15.6 particles/g, and the lowest was zero.

### 3.3. Characteristics of Microplastics in Placenta, Cord Blood, and Meconium

**Figure 1:**
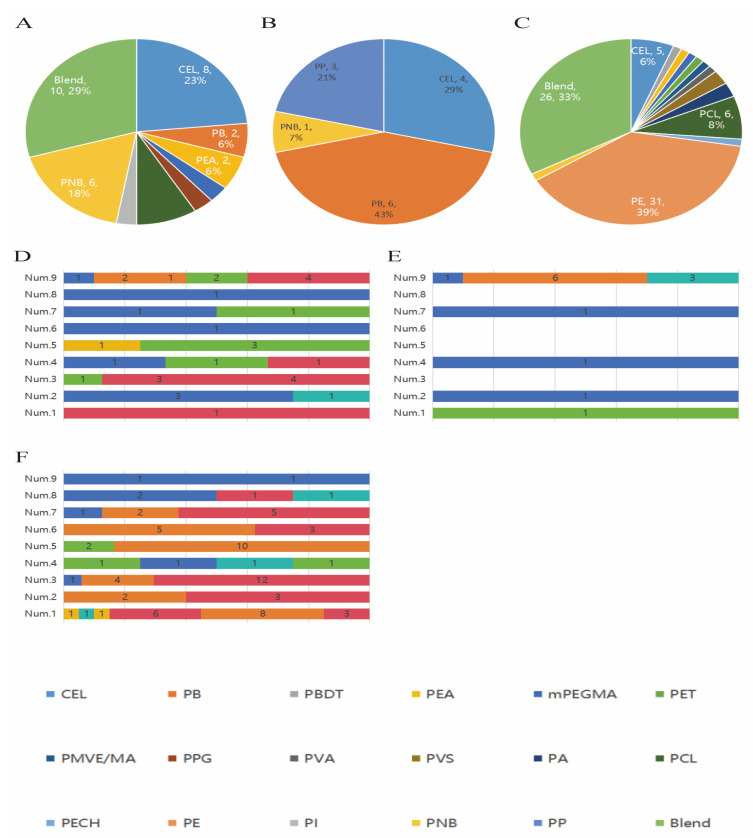
Relative frequencies of different types of MPs in placenta (**A**), cord blood (**B**), and meconium (**C**); distribution of MPs in placenta (**D**), cord blood (**E**), and meconium (**F**) in 9 participants. Note: The numbers in the images represent the count of detected MPs. Blend: Microplastic blends (merging similar types of plastics, concluding PDADMAC, poly dimer acid-co-alkyl polyamine; P(EA-co-MAA-co-2-IEM), poly ethyl acrylate-co-methacrylic acid-co-3-(1-isocyanato-2-methyl ethyl); P(EA-co-MAA-co-1-IEM), poly ethyl acrylate-co-methacrylic acid-co-3-(1-isocyanato-1-methyl ethyl); PEMG, poly ethylene-co methyl acrylate-co-glycidyl methacrylate; PEB, poly ethylene-co-1-butene; PEB-2, poly ethylene-co-2-butene; EAA, poly ethylene-co-acrylic acid; EEA, poly ethylene-co-ethyl acrylate; POE, poly ethylene-co-octene; EVA, poly ethylene-co-vinyl acetate; PLMA-EDMA, poly lauryl mathacrylate-co-ethylene glycol dimethacrylate; PVP/VA, poly N-vinylpyrrolidone-co-vinyl acetate; PBE, poly propylene-co-1-butene-co-ethylene; PVAL/E, poly vinyl alcohol-co-ethylene; and PDMS-co-HEMA, polydimethylsiloxane-co-3-2-2-hydroxyethexy ethexy). Abbreviations: CEL, microplastic cellulose; PB, poly butene isotactic; PBDT, poly butadiene phenyl terminated; PEA, poly ethylene adipate; mPEGMA, poly ethylene glycol ehtylether methacrylate; PET, poly ethylene terephthlate; PMVE/MA, poly methyl vinyl ether-maleic; PPG, poly propylene glycol; PVA, poly vinyl alcohol; PVS, poly vinyl stearate; PA, polyamide resin; PCL, polycaprolactone; PECH, polyepichlorohydrin; PE, polyethylene; PI, polyisoprene hydrogenated; PNB, polynorbornene; PP, polypropylene.

The distribution of types and frequencies of total MPs identified in placenta, cord blood, and meconium is shown in Figure 1A–C. The characteristics of MPs identified in the placenta, cord blood, and meconium of each participant are shown in Figure 1D–F. Corresponding numerical data are reported in Table S2a,b.

Nine types of MPs were identified in the placenta. The highest abundance was CEL, followed by PNB, which accounted for 23.5% (8/34) and 17.6% (6/34), respectively. Four types of MPs were identified in cord blood, and the highest abundance was for PB, accounting for 42.9% (6/14). Fourteen types of MPs were identified in the meconium. The highest abundance was PE, followed by Blend and PCL, which accounted for 38.8% (31/80), 32.5% (26/80), and 7.5% (6/80), respectively.

The characteristics of MPs in the placenta, cord blood, and meconium varied among participants. In the placenta, the frequency of MPs varied from 1 to 10 particles, with a median frequency of 3 particles. The highest number of MPs was found for the ninth sample in the cord blood, with a total of 10 MP particles. Six types of MPs were identified in the first meconium sample, with a total of 20 MP particles, among which 8 PE particles were detected.

### 3.4. Particle Size and Color

**Figure 2:**
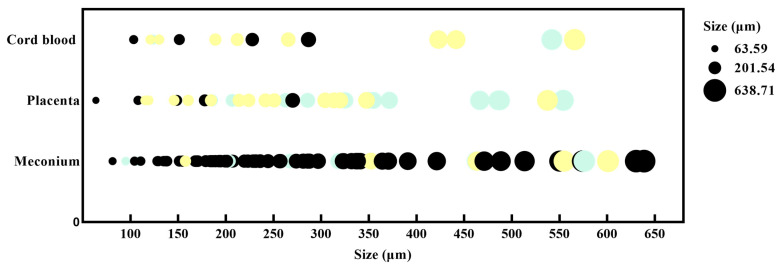
The distribution of the size and color of MP particles in the placenta, cord blood, and meconium (blue stands for transparent, black stands for black, and yellow stands for yellow).

The colors and particle sizes of MPs in the placenta, cord blood, and meconium are shown in Figure 2. Corresponding numerical data are reported in Table S3.

The colors of MPs in the placenta were mainly yellow and transparent (blue color represents transparent color in Figure 2). MPs in cord blood were mainly yellow, and MPs in meconium were mainly black. The sizes of MPs varied among different types of samples, with >80% of the MPs ranging from 100 to 400 μm.

All MPs were in the shape of fragments. Photographs of MPs pictured by micro-Raman spectra are shown in Figure S1.

### 3.5. Associations Between the Abundance of Different Microplastics in the Placenta, Cord Blood, and Meconium

**Figure 3:**
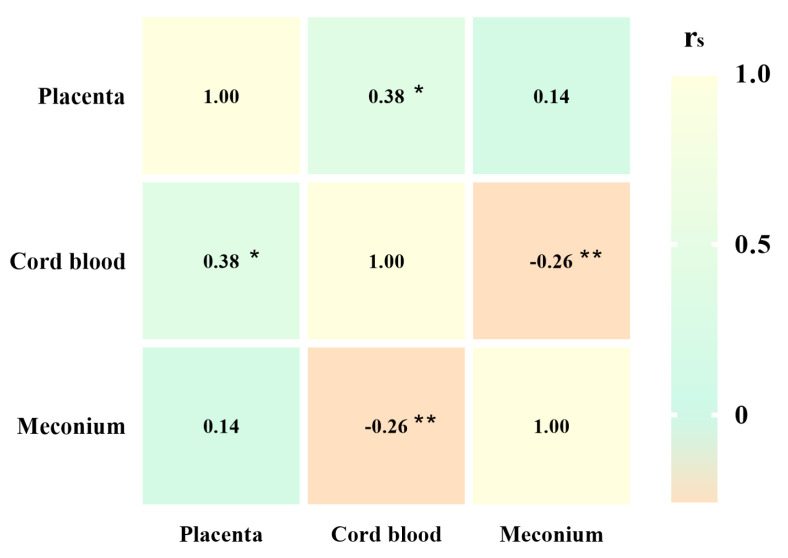
Different types of MPs abundance correlation analysis in placenta, cord blood, and meconium (*: *p* < 0.05; **: *p* < 0.01).

The relationships of different types of MPs with the placenta, cord blood, and meconium are shown in Figure 3. The abundance of different types of MPs in meconium was negatively correlated with that in cord blood (rs = −0.26, *p* < 0.01). The abundance of different types of MPs in cord blood was positively correlated with that in the placenta (rs = 0.38, *p* < 0.05).

**Figure 4:**
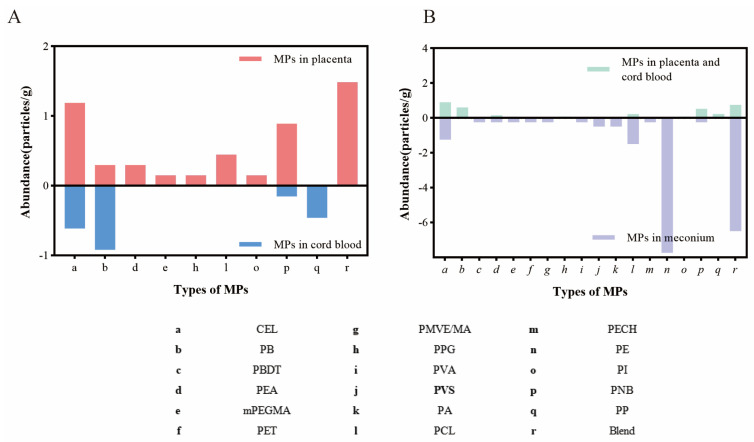
(**A**) The comparison of the abundance of MPs between placenta and cord blood. (**B**) The comparison of the abundance of MPs between placenta, cord blood, and meconium.

A comparison of MPs in the placenta and cord blood is shown in Figure 4A. The results showed that three types of MPs, CEL, PB, and PNB, were identified both in placenta and cord blood. A comparison of MPs in placenta, cord blood, and meconium is shown in Figure 4B. The results showed that the abundance and type of MPs were different, and six types of MPs were found in all of them.

### 3.6. Relationship of the Presence of MPs in Placenta, Cord Blood, and Meconium Stratified by Living Environment and Lifestyles During Pregnancy

Table 3 shows the presence of MPs in the placenta, cord blood, and meconium, according to the living environment and lifestyle during pregnancy. The abundance of MPs in meconium from participants who drank tea ≥ 3 times/week during pregnancy was lower than those who drank tea less than 3 times/week (*p* = 0.048). Though not statistically significant, we observed that the abundance of MPs in placenta and cord blood from women who often used plastic bottles/cups for drinking was higher than that in those who seldom used these or did not. There was no statistically significant difference in the abundance of different MPs in the placenta and cord blood according to other habits.

## 4. Discussion

Our study presents evidence of MP transfer via the placenta–cord blood–meconium pathway. We also strengthen the evidence for the presence of MPs in human cord blood. The highest abundance of MPs was in meconium, followed by placenta and cord blood. The abundance of different types of MPs in cord blood was positively correlated with that in placenta. When combining questionnaire information about the living environment and lifestyle during pregnancy, we observed that the habit of drinking tea less than 3 times per week was associated with a lower level of MP exposure in meconium.

Few studies have compared the relationship between the abundances of MPs in the placenta, cord blood, and meconium. We compared the abundances of different types of MPs in the placenta, cord blood, and meconium and found that there was a positive correlation between placenta and cord blood (rs = 0.38, *p* < 0.05). The distribution of MPs in the placenta and umbilical cord blood indicates that CEL, PB, and PNB can be detected in both of them, suggesting that some specific types of MPs may have the potential to penetrate the placental barrier and enter the umbilical cord blood, thereby directly exposing the fetus. Meanwhile, the presence of MPs in meconium samples further provides potential evidence that MPs can be transferred via the placenta–cord blood–meconium pathway. To our knowledge, no studies have reported evidence of MP transfer via this pathway. However, more research is needed to support the hypothesis.

The presence of MPs has been found in human blood [42]. A study identifying the presence of MPs in the thrombus found that the number of MP particles in the thrombus was significantly positively correlated with the blood platelet level [48], which indicates that MP exposure poses a threat to human health. Recently, a study [54] confirmed the presence of MPs in human umbilical vein blood, consistent with our research findings. However, while the main type of MP detected in umbilical vein blood in that study was PA, our study predominantly identified PB, which is inconsistent with their findings. The differences may be due to the varying participants in different regions. Our study further strengthens the evidence on MPs in human cord blood. Interestingly, one participant was found to have a higher number of particles in the cord blood than others, with a total of ten MP particles, where the highest abundance was of PB. This may be attributed to the impact of medical procedures. A study found that the types and diameter distribution of MPs in the blood showed alterations following a surgical procedure [37], indicating that medical procedures may be a path of MP exposure. During cesarean surgery, the participant may be exposed to various medical devices in plastic, such as the protective film of surgical incision, syringe barrel, etc., which may be a potential factor for exposure to MPs. Moreover, due to being diagnosed with preterm rupture of membranes, the participant stayed in the hospital for several days, which might directly and indirectly increase the exposure to MPs in the circulatory system via medical operations (e.g., intravenous injection) and the use of single-use plastic products during the hospitalization period [55,56]. Previous studies showed that PB is widely used in tubing, film, medical and pharmaceutical packaging, etc. [57], which is consistent with our results. Given the difficulty of collecting blood samples (e.g., blood) from newborns, umbilical cord blood is considered an effective biological matrix for reflecting an infant’s exposure to environmental pollutants, such as per- and poly-fluoroalkyl substances (PFASs) [58]. Therefore, although the abundance of MPs in cord blood was not significant in our study, the fetus is extremely sensitive to contaminants even with small doses, so paying attention to MP exposure and infant health is a must. And the result needs to be verified by more studies in the future.

In our results, we found that the abundance of MPs in meconium was higher than those in placenta and cord blood, consistent with the findings of Liu et al. [46], which may be attributed to the following reasons. First, an accumulative effect. Meconium is made up of a variety of substances from the digestive tract, mainly saliva, gastric juices, and fetal hair; it accumulates continuously in the fetus from 16 weeks of gestation and is not excreted until delivery [59]. Several studies have confirmed that MPs can be found in placenta and amniotic fluid [38,60,61]. Considering the physiological functions of the placenta and amniotic fluid, the abundance of MPs within them may influence the abundance of MPs in meconium. Second, MPs are ubiquitous and everything has the potential to be contaminated. Although we strictly followed the sampling protocols established in the existing literature to minimize contact between the meconium and diapers, the possibility of meconium contamination by MPs during the collection process cannot be completely ruled out. Definitively, PP, which is commonly used in the surface materials of diapers, was not identified in meconium [62], indicating good quality control in sampling collection. However, the potential factors affecting MP exposure in newborns remain unclear, and more relevant cohort studies should be carried out to clarify the exposure factors.

The type of detectable MP varies in different biological samples. The predominant type of MP identified in sputum was polyurethane (PU) [43], in blood were PET and PE [42], in cardiac tissue was polyethylene terephthalate (PET) [37], and in the endometrium were EAA and PE [40]. In our results, the predominant type of MP in meconium was PE, which accounted for 38.8% (31/80). These results are consistent with the fact that PE is currently the plastic with the highest annual production worldwide [63]. Interestingly, we found different types of MPs in the placenta, umbilical cord blood, and meconium. There are many possible reasons. Firstly, differentiation in sources of exposure. A study found that MPs can be transferred within the body [64], and the presence of MPs in meconium may be the result of maternal–fetal transfer. The swallowing of amniotic fluid by the fetus in utero could be a significant pathway for the transfer of MPs. Notably, our results found that the types of MPs in meconium were the most diverse, which may be the result of the fetus’s long-term swallowing of amniotic fluid, as we know that fetuses start to swallow from the 18th week of gestation. Moreover, the placenta and umbilical cord are structures facilitating the exchange of substances and gases between the mother and fetus. Their exposure to MPs may primarily originate from the maternal circulatory system. In addition, the lifestyle contributes to disparities in MP exposure. MPs are widespread and have been found in dust, water, seafood, salt, honey, fruits, vegetables, cosmetics, toothpaste, and so on. Different dietary habits result in different concentrations of MPs in biological samples. Consequently, the abundance of MP exposure varies among individuals.

The size of MP particles is an important factor in health effects. Several animal studies showed that the size of MP particles is a major factor affecting placental function [17,65]. In addition, previous studies have indicated that regardless of their chemical composition, MPs can elicit immune-inflammatory responses, with particle size being a primary factor. Interestingly, contrary to the common assumption that smaller particles have a greater impact on health [66,67], there is evidence suggesting that larger particles (75–200 μm) significantly enhance the inflammatory expression of immune cells [68]. The size of MP particles ranged from 63.59 to 638.71 μm in our study, with ≤400 μm accounting for 80.47%, which seems to be relatively larger than other research. However, MP particle sizes were found to vary among different types of samples. A study showed that the size of MP particles ranges from 20 to 800 μm [45] in adult feces. Another study found an average size of 223.10 ± 436.16 μm (range between 12 and 2475 μm) was identified [36] in human lung tissue. Nanometer-sized MP particles can penetrate the placental barrier and enter the fetus, as confirmed in previous studies [60], but the evidence for micrometer-sized particles is still insufficient, and relevant studies need to be performed to explore the transport mechanism in the future.

MPs are primarily exposed through the gastrointestinal tract [69,70,71], but fewer studies investigate the influence of MP exposure according to the lifestyles during pregnancy on the abundance of MPs in the placenta, umbilical cord blood, and meconium. Liu et al. [46] found that drinking water and use of scrubs or toothpaste may be a source of MP exposure in pregnant women. Breastfeeding, bottles, and plastic toys may be sources of MP exposure in infants. However, another study found no statistically significant association between lifestyle factors and the abundance of MPs [54]. In our study, the abundance of MPs in meconium from newborns who were born to mothers with tea-drinking habits ≥ 3 times/week was lower as compared to those who drank tea < 3 times/week. Previous studies have indicated the presence of MPs [72] in tea bags, and the more frequent the consumption of tea, the greater the exposure to MPs. On the contrary, our results are inconsistent with them. Water sources may play an important role. A study found that during the process of boiling water, nanoplastics can transfer from the water to the scale deposits in the pot, thereby reducing the MPs in the water, which decreases the ingestion of MPs to some extent [73]. Furthermore, tea consumption aids digestion and may contribute to the elimination of MPs from the body. Additionally, MPs are ubiquitous, with tea consumption being just one of the exposure pathways. More research is needed to support and clarify the hypothesis.

Adding to the current body of knowledge, our study presents evidence of MP transfer via the placenta–cord blood–meconium pathway, offering new insights into the direct exposure of neonates to MPs. Additionally, our study strengthens the evidence for the presence of MPs in human cord blood. MP exposure affects umbilical artery blood flow, which is associated with various adverse pregnancy outcomes. Further research should be conducted to elucidate the impact of MP exposure in umbilical cord blood on pregnancy health.

However, certain limitations should be noted. Firstly, the sample size of our study is relatively small, which makes it challenging to extrapolate the potential exposure routes of MPs. Therefore, more research with larger sample sizes should be conducted. Secondly, while the questionnaire is designed to investigate lifestyle habits and dietary intake, it may not be comprehensive enough. To better assess the sources of MP exposure, more detailed and comprehensive questionnaires should be designed to quantitatively assess the frequency of plastic product usage. Thirdly, the impact of pretreatment methods before detection is significant. Digestion with hydrogen peroxide and nitric acid might affect the surface of MPs, causing the quantities to be underestimated. Fourthly, there are limitations to detection methods. Although micro-Raman spectroscopy is widely used for the identification of MPs in biological samples [48,49,50,74], it has certain limitations. These include requirements for the particle size of MPs, susceptibility to fluorescence interference, high detection costs, and extended detection times. These factors, to some extent, limit its application in the observation of MPs in human tissues. Nonetheless, it is worth affirming that Raman spectroscopy detection technology has the advantages of high spatial resolution and being unaffected by moisture, allowing it to simultaneously collect information on the size, color, and surface morphology of MP particles. Compared to micro-Raman spectroscopy, FTIR is not affected by fluorescence interference, but it requires a flat sample surface and is susceptible to moisture interference. In summary, there are certain shortcomings in quantifying and identifying MP particles using a single method. To compensate for these limitations in future research, we need to combine different technologies for the identification of MPs to improve the accuracy and efficiency of their identification. Lastly, while we focused on MP exposure in the placenta, cord blood, and meconium, the exact microparticle sources and the specific impact of MPs on newborns’ health are not yet clear.

## 5. Conclusions

Our study presents evidence of MP transfer via the placenta–cord blood–meconium pathway. We also strengthen the evidence for the presence of MPs in human cord blood. The abundance of MPs exhibited the order of meconium > placenta > cord blood. We also observed that dietary habits during pregnancy were associated with MP exposure in meconium. More studies should be conducted to clarify the relationship between lifestyles during pregnancy and MP exposure in the placenta, umbilical cord blood, amniotic fluid, and meconium, and to explore the potential health effects of MP exposure.

## Figures and Tables

**Table 1:** Demographic characteristics of the pregnant women and newborns in this study (*n* = 9).

Characteristics	Categories	N (%)
BMI before pregnancy (kg/m^2^), median (IQR)	-	21.3 (18.7, 23.8)
Age (year), median (IQR)	-	31.8 (28.5, 37.0)
Education	High school and below	1 (11.1)
	Bachelor’s degree	7 (77.8)
	Postgraduate and above	1 (11.1)
Occupation	Governmental official	0 (0.0)
	Profession and technology	1 (11.1)
	General office	4 (44.4)
	Business services	3 (33.3)
	Others	1 (11.1)
Annual income per capita (CNY)	<100 thousand	4 (44.4)
	100–200 thousand	3 (33.3)
	>200 thousand	2 (22.2)
Parity	Primipara	4 (44.4)
	Multipara	5 (55.6)
Mode of conception	Natural conception	9 (100.0)
	Assisted reproduction	0 (0.0)
Birth outcome	Preterm	4 (44.4)
	Full-term	5 (55.6)
Mode of delivery	Vaginal delivery	7 (77.8)
	Cesarean delivery	2 (22.2)
Sex of newborns	Male	5 (55.6)
	Female	4 (44.4)
Newborn weight (g), median (IQR)	-	2980 (2200, 3210)

Abbreviation: BMI: body mass index; N (%): numbers and percentages; IQR: interquartile range.

**Table 2:** The abundance of MPs in the placenta, cord blood, and meconium of 9 participants.

Participants	Frequency of Microplastic Particles (Particle)			Samples’ Dry Weight (g)			Abundance (Particle/g)		
	Placenta	Cord Blood	Meconium	Placenta	Cord Blood	Meconium	Placenta	Cord Blood	Meconium
Num.1	1	1	20	0.4381	0.9058	0.3076	2.28	1.10	65.02
Num.2	4	1	5	0.8032	0.6789	0.2410	4.98	1.47	20.75
Num.3	8	-	17	0.8740	0.4166	0.2203	9.15	0	77.17
Num.4	3	1	4	0.9531	0.7687	0.7481	3.15	1.30	5.35
Num.5	4	-	12	0.6093	0.8135	0.7527	6.56	0	15.94
Num.6	1	-	8	0.8073	0.8213	0.2224	1.24	0	35.98
Num.7	2	1	8	0.7689	0.6111	0.1221	2.60	1.63	65.52
Num.8	1	-	4	0.7342	0.8570	0.4901	1.37	0	8.16
Num.9	10	10	2	0.7459	0.6409	0.8963	13.41	15.60	2.23
Median (Interquartile range)	3 (1, 6)	1 (0, 1)	8 (4, 15)	-	-	-	3.15 (1.82, 7.85)	1.1 (0, 1.55)	20.75 (6.75, 65.27)
Total	34	14	80	-	-	-	-	-	-
*P* ^a^	-	-	-	-	-	-	0.001 ^b^		

^a^: Kruskal–Wallis H test (two-tail). ^b^: *p* < 0.05.

**Table 3:** The presence of MPs in the placenta, cord blood, and meconium according to the living environment and lifestyle during pregnancy.

	Questions	Options	N (%)	Placenta	Cord Blood	Meconium
Living environment	Having sites of air pollution around your home or not	no	8 (88.9)	2.9 (1.6, 8.1)	1.2 (0, 1.6)	28.4 (6.1, 65.4)
		yes	1 (11.1)	-	-	-
	*P* ^a^			0.667	0.444	0.889
	Distance of residence from major transportation arteries (two-way, four-lane roads)	≤100 m	5 (55.6)	2.3 (1.3, 8.0)	1.1 (0, 8.6)	36.0 (5.2, 65.3)
		>100 m	4 (44.6)	5.8 (3.6, 8.5)	0.6 (0, 1.4)	18.3 (8.0, 63.1)
	*P* ^a^			0.190	0.730	1.000
	Frequency of using air purifiers at home	never/seldom	8 (88.9)	4.1 (1.6, 8.5)	0.5 (0, 1.4)	18.3 (6.1, 57.8)
		often	1 (11.1)	-	-	-
	*P* ^a^			0.889	0.444	0.444
Dietary and lifestyle during pregnancy	Drinking water	piped water	5 (55.6)	5.0 (2.7, 11.3)	1.3 (0.5, 8.5)	20.8 (3.8, 71.1)
		barrel-loaded water	4 (44.6)	2.0 (1.3, 5.6)	0 (0, 1.2)	26.0 (10.1, 58.1)
	*P* ^a^			0.190	0.286	1.000
	Passive smoking	no	7 (77.8)	5.0 (2.3, 9.1)	1.1 (0, 1.6)	20.7 (8.2, 65.5)
		yes	2 (22.2)	2.2 (1.2, 3.1)	0.6 (0, 1.3)	20.7 (5.3, 36.0)
	*P* ^a^			0.333	0.667	0.667
	Drinking water in plastic cup/bottles	never/seldom	7 (77.8)	3.1 (2.3, 6.6)	1.1 (0, 1.5)	36.0 (15.9, 65.5)
		often	2 (22.2)	7.4 (1.4, 13.4)	7.8 (0, 15.6)	5.2 (2.2, 8.2)
	*P* ^a^			1.000	0.667	0.111
	Milk tea consumption	never/seldom	9 (100.0)	3.1 (1.8, 7.9)	1.1 (0, 1.5)	20.7 (6.8, 65.3)
		often	0 (0.0)	-	-	-
	*P* ^a^			-	-	-
	Tea consumption	never/seldom	6 (66.7)	2.4 (1.3, 6.0)	0.5 (0, 1.5)	50.5 (17.6, 68.4)
		often	3 (33.3)	6.6 (3.1, 13.4)	1.3 (0, 15.6)	5.3 (2.2, 15.9)
	*P* ^a^			0.167	0.548	0.048 ^b^
	Drinking beverages in plastic bottles	never/seldom	9 (100.0)	3.1 (1.8, 7.9)	1.1 (0, 1.5)	20.7 (6.8, 65.3)
		often	0 (0.0)	-	-	-
	*P* ^a^			-	-	-
	Microwave food using plastic tableware	never/seldom	8 (88.9)	2.9 (1.6, 6.2)	1.2 (0, 1.6)	18.3 (6.1, 57.8)
		often	1 (11.1)	-	-	-
	*P* ^a^			0.444	0.444	0.222
Dietary and lifestyle during pregnancy	Takeout frequency	never/seldom	9 (100.0)	3.1 (1.8, 7.9)	1.1 (0, 1.5)	20.7 (6.8, 65.3)
		often	0 (0.0)	-	-	-
	*P* ^a^			-	-	-
	Eating seafood (fish, shellfish)	never/seldom	5 (55.6)	5.0 (1.3, 10.0)	0 (0, 8.5)	15.9 (5.2, 28.4)
		often	4 (44.6)	2.9 (2.4, 7.6)	1.2 (0.3, 1.5)	65.3 (20.3, 74.3)
	*P* ^a^			1.000	0.730	0.190
	Time for daily wear of the mask	<3 h/day	6 (66.7)	3.8 (1.3, 7.2)	0 (0, 1.5)	28.4 (14.0, 68.4)
		≥3 h/day	3 (33.3)	3.1 (2.3, 13.4)	1.3 (1.1, 15.6)	5.3 (2.2, 65.0)
	*P* ^a^			0.714	0.262	0.262
	Drinking boxed milk	never/seldom	1 (11.1)	-	-	-
		often	8 (88.9)	2.9 (1.6, 8.1)	1.2 (0, 1.6)	28.4 (6.1, 65.4)
	*P* ^a^			0.667	0.444	1.000
	Scrub cleanser	never/seldom	1 (11.1)	-	-	-
		often	8 (88.9)	4.1 (2.4, 8.5)	1.2 (0, 1.6)	18.3 (6.1, 65.4)
	*P* ^a^			0.222	0.444	0.889
	Toothpaste	<3 times/day	7 (77.8)	3.1 (1.4, 6.6)	1.3 (0, 1.6)	15.9 (5.3, 36.0)
		≥3 times/day	2 (22.2)	5.7 (2.3, 9.1)	0.5 (0, 1.1)	71.1 (65.0, 77.2)
	*P* ^a^			1.000	0.500	0.111

Note: Seldom: <3 times/week; often: ≥3 times/week. Abbreviation: N (%): numbers and percentages. ^a^: Mann–Whitney U test (two-tail). ^b^: *p* < 0.05.

## Data Availability

Data will be made available on request.

## Supplementary Materials

The following supporting information can be downloaded at https://www.mdpi.com/article/10.3390/toxics12120850/s1, Table S1: Questionnaire for pregnant women, including baseline information and information about their lifestyle. Table S2: (a) Total number of MPs detected in placenta, cord blood, and meconium samples; (b) number of MPs detected in placenta, cord blood, and fecal samples among nine different participants. Table S3: Particle size and color distribution of MP particles in the placenta, cord blood, and meconium samples. Figure S1: Photographs and Raman spectra to demonstrate microplastics detected in meconium samples. Figures S2–S5: Certificates of identification for potential microplastic particle species in our study.
