# Supplementary material for: Microplastic Particles Detected in Fetal Cord Blood, Placenta, and Meconium: A Pilot Study of Nine Mother–Infant Pairs in South China
Source: Toxics. 2024 Nov 26;12(12):850. doi: 10.3390/toxics12120850 (PMC11679465; doi:10.3390/toxics12120850)
Supplement: Supplementary file 1 [file toxics-12-00850-s001.zip › toxics-3269958-supplementary.pdf]

Supplementary material

Microplastic Particles Detected in Fetal Cord Blood, Placenta, and Meconium: A Pilot Study of Nine Mother–Infant Pairs in South China

Contents

Text S1 and corresponding **Table S1**.

Text S2 and corresponding **Table S2(a) and Table S2(b)**.

Text S3 and corresponding **Table S3(a) and Table S3(b)**.

Text S4 and corresponding **Figure S1**.

Text S5 and corresponding **Figure S2 - Figure S5.**

Supplementary text

Text S1. Table S1: Questionnaire for pregnant women, including baseline information and information about their lifestyle.

Text S2. Table S2 (a): Total number of microplastics detected in placenta, cord blood, and meconium samples, respectively; (b) number of microplastics detected in placenta, cord blood, and fecal samples among nine different participants.

Text S3. Table S3: Particle size and color distribution of MP particles in the placenta, cord blood, and meconium samples.

Text S4. Figure S1: Photographs and Raman spectra to demonstrate microplastics detected in meconium samples.

Text S5. Figures S2 - S5: Certificates of identification for potential microplastic particle species in our study.

Supplemental Table S1 Questionnaire

Questions	Options
**Basic information**	
Age (year)	-
Ethnicity	-
Height (cm)	-
Weight before pregnancy (kg)	-
Education	High school and below; Bachelor's degree ; Postgraduate and above
Parity	primipara; multipara
Annual income per capita (CNY)	<100 thousand; 100-200 thousand; >200 thousand
Occupation	Governmental official; Profession and technology; General office; Business services; others
Mode of conception	Natural conception; Assisted reproduction
**Lifestyle**	
Having sites of air pollution around your home or not	no; yes
Distance of residence from major transportation arteries (two-way, four-lane roads)	≤100 m; >100 m
Frequency of using air purifiers at home	never/seldom/often
Drinking water	piped water; barrel-loaded water
Passive smoking	no; yes
Drinking water in plastic cups/bottles	never/seldom/often
Milktea consumption	never/seldom/often
Tea consumption	never/seldom/often
Drinking beverages in plastic bottles	never/seldom/often
Microwave food using plastic tableware	never/seldom/often
Takeout frequency	never/seldom/often
Eating seafood(fish,shellfish)	never/seldom/often
Time for daily wear of the mask	<3 hours/day; ≥3 hours/day
Drinking boxed milk	never/seldom/often
Scrub cleanser	never/seldom/often
Toothpaste	<3 times/day; ≥ 3times/day

Note: Seldom: < 3 times/week; often: ≥ 3 times/week.

Supplemental Table S2(a) Frequency of different types of microplastics in the placenta, cord blood and meconium

Type of MPs	Frequency of MPs in different types of samples		
	Placenta	Cord blood	Meconium
CEL	8	4	5
PB	2	6	-
PBDT	-	-	1
PEA	2	-	1
mPEGMA	1	-	1
PET	-	-	1
PMVE/MA	-	-	1
PPG	1	-	-
PVA	-	-	1
PVS	-	-	2
PA	-	-	2
PCL	3	-	6
PECH	-	-	1
PE	-	-	31
PI	1	-	-
PNB	6	1	1
PP	-	3	-
Blend	10	-	26

Note: Blend: Microplastic blends (Include: PDADMAC, poly dimer acid-co-alkyl polyamine; P(EA-co-MAA-co-2-IEM), poly ethyl acrylate-co-methacrylic acid-co-3-(1-isocyanato-2-methyl ethyl); P(EA-co-MAA-co-1-IEM), poly ethyl acrylate-co-methacrylic acid-co-3-(1-isocyanato-1-methyl ethyl); PEMG, poly ethylene-co methyl acrylate-co-glycidyl methacrylate; PEB, poly ethylene-co-1-butene;PEB-2, poly ethylene-co-2-butene; EAA, poly ethylene-co-acrylic acid; EEA, poly ethylene-co-ethyl acrylate; POE, poly ethylene-co-octene; EVA, poly ethylene-co-vinyl acetate; PLMA-EDMA, poly lauryl mathacrylate-co-ethylene glycol dimethacrylate; PVP/VA, poly N-vinylpyrrolidone-co-vinyl acetate; PBE, poly propylene-co-1-butene-co-ethylene; PVAL/E, poly vinyl alcohol-co-ethylene; and PDMS-co-HEMA, polydimethylsiloxane-co-3-2-2-hydroxyethexy ethexy). **Abbreviations:** CEL, microplastic cellulose; PB,poly butene isotactic; PBDT, poly butadiene phenyl terminated; PEA, poly ethylene adipate; mPEGMA, poly ethylene glycol ehtylether methacrylate; PET, poly ethylene terephthlate ; PMVE/MA, poly methyl vinyl ether-maleic; PPG, poly propylene glycol; PVA, poly vinyl alcohol; PVS, poly vinyl stearate; PA, polyamide resin; PCL, polycaprolactone; PECH, polyepichlorohydrin; PE, polyethylene; PI, polyisoprene hydrogenated; PNB, polynorbornene; PP, polypropylene.

Supplemental Table S2(b) Frequency of different types of microplastics in the placenta, cord blood and meconium among different individuals

Participants	Types of samples	Types of microplastics	Frequency(particles)
Num.1	placenta	Blend	1
	cord blood	PNB	1
	meconium	PBDT	1
		mPEGMA	1
		Blend	3
		PVA	1
		PCL	6
		PE	8
Num.2	placenta	CEL	3
		mPEGMA	1
	cord blood	CEL	1
	meconium	Blend	3
		PE	2
Num.3	placenta	PEA	1
		Blend	4
		PCL	3
	cord blood	-	-
	meconium	CEL	1
		Blend	12
		PE	4
Num.4	placenta	CEL	1
		PEA	1
		Blend	1
	cord blood	CEL	1
	meconium	PEA	1
		PMVE/MA	1
		PA	1
		PNB	1

[continued]

Supplemental Table S2(b), continued.

Participants	Types of samples	Types of microplastics	Frequency(particles)
Num.5	placenta	PNB	3
		PI	1
	cord blood	-	-
	meconium	PVS	2
		PE	10
Num.6	placenta	CEL	1
	cord blood	-	-
	meconium	Blend	3
		PE	5
Num.7	placenta	CEL	1
		PNB	1
	cord blood	CEL	1
	meconium	CEL	1
		Blend	5
		PE	2
Num.8	placenta	CEL	1
	cord blood	-	-
	meconium	CEL	2
		PET	1
		PA	1
Num.9	placenta	CEL	1
		PNB	2
		PB	2
		Blend	4
		PPG	1
	cord blood	CEL	1
		PB	6
		PP	3
	meconium	CEL	1
		PECH	1

Note: Blend: Microplastic blends (Include: PDADMAC, poly dimer acid-co-alkyl polyamine; P(EA-co-MAA-co-2-IEM), poly ethyl acrylate-co-methacrylic acid-co-3-(1-isocyanato-2-methyl ethyl); P(EA-co-MAA-co-1-IEM), poly ethyl acrylate-co-methacrylic acid-co-3-(1-isocyanato-1-methyl ethyl); PEMG, poly ethylene-co methyl acrylate-co-glycidyl methacrylate; PEB, poly ethylene-co-1-butene;PEB-2, poly ethylene-co-2-butene; EAA, poly ethylene-co-acrylic acid; EEA, poly ethylene-co-ethyl acrylate; POE, poly ethylene-co-octene; EVA, poly ethylene-co-vinyl acetate; PLMA-EDMA, poly lauryl mathacrylate-co-ethylene glycol dimethacrylate; PVP/VA, poly N-vinylpyrrolidone-co-vinyl acetate; PBE, poly propylene-co-1-butene-co-ethylene; PVAL/E, poly vinyl alcohol-co-ethylene; and PDMS-co-HEMA, polydimethylsiloxane-co-3-2-2-hydroxyethexy ethexy). **Abbreviations:** CEL, microplastic cellulose; PB,poly butene isotactic; PBDT, poly butadiene phenyl terminated; PEA, poly ethylene adipate; mPEGMA, poly ethylene glycol ehtylether methacrylate; PET, poly ethylene terephthlate ; PMVE/MA, poly methyl vinyl ether-maleic; PPG, poly propylene glycol; PVA, poly vinyl alcohol; PVS, poly vinyl stearate; PA, polyamide resin; PCL, polycaprolactone; PECH, polyepichlorohydrin; PE, polyethylene; PI, polyisoprene hydrogenated; PNB, polynorbornene; PP, polypropylene.

Supplemental Table S3 **Distribution of particle size and color of microplastic particles in different samples**

Meconium			Placenta			Cord blood		
Types of microplastics	Size(um)	Colour	Types of microplastics	Size(um)	Colour	Types of microplastics	Size(um)	Colour
poly methyl vinyl ether-maleic	267.28	transparent	poly ethylene adipate	185.25	transparent	cellulose	151.15	black
polynorbornene	94.93	transparent	cellulose	206.45	transparent	polynorbornene	123.50	transparent
poly ethylene adipate	168.66	transparent	poly lauryl mathacrylate-co-ethylene glycol dimethacrylate	466.36	transparent	cellulose	541.94	transparent
polyamide resin	225.81	transparent	poly lauryl mathacrylate-co-ethylene glycol dimethacrylate	354.84	transparent	cellulose	265.44	yellow
poly propylene-co-1-butene-co-ethylene	184.33	black	poly ethylene-co methyl acrylate-co-glycidyl methacrylate	485.71	transparent	poly butene isotactic	129.95	yellow
poly propylene-co-1-butene-co-ethylene	134.56	black	polycaprolactone	487.56	transparent	polypropylene	441.47	yellow
poly propylene-co-1-butene-co-ethylene	199.08	black	polycaprolactone	285.71	transparent	poly butene isotactic	565.90	yellow
poly propylene-co-1-butene-co-ethylene	370.51	black	poly ethylene adipate	324.42	transparent	polypropylene	188.94	yellow
polyethylene	638.71	black	poly ethylene-co methyl acrylate-co-glycidyl methacrylate	350.23	transparent	poly butene isotactic	423.04	yellow
poly ethylene-co-octene	550.23	black	poly lauryl mathacrylate-co-ethylene glycol dimethacrylate	324.42	transparent	poly butene isotactic	211.98	yellow
poly ethylene-co-1-butene	206.45	black	polycaprolactone	371.43	transparent	poly butene isotactic	103.23	black
poly dimer acid-co-alkyl polyamine	285.71	black	cellulose	241.47	yellow	polypropylene	120.74	yellow

[continued]

Supplemental Table S3, continued.

Meconium			Placenta			Cord blood		
Types of microplastics	Size(um)	Colour	Types of microplastics	Size(um)	Colour	Types of microplastics	Size(um)	Colour
poly ethylene-co-1-butene	219.35	black	poly ethylene-co-vinyl acetate	63.59	black	poly butene isotactic	286.64	black
cellulose	341.01	black	poly ethylene glycol ethylether methacrylate	325.35	transparent	cellulose	227.65	black
polyethylene	202.76	black	cellulose	148.39	black			
poly propylene-co-1-butene-co-ethylene	256.22	black	cellulose	107.83	black			
polyethylene	390.78	black	cellulose	177.88	black			
poly ethylene-co-1-butene	231.34	black	cellulose	553.92	transparent			
polyethylene	154.84	black	poly ethyl acrylate-co-methacrylic acid-co-3-(1-isocyanato-1-methyl ethyl)-	537.33	yellow			
poly propylene-co-1-butene-co-ethylene	207.37	black	poly propylene glycol	262.67	transparent			
poly ethylene-co-1-butene	201.84	black	poly ethyl acrylate-co-methacrylic acid-co-3-(1-isocyanato-1-methyl ethyl)-	213.82	yellow			
poly ethylene-co-ethyl acrylate	317.97	transparent	poly ethyl acrylate-co-methacrylic acid-co-3-(1-isocyanato-2-methyl ethyl)-	347.47	yellow			
polyethylene	462.67	transparent	polynorbornene	223.96	yellow			
polyethylene	137.33	black	poly butene isotactic	304.15	yellow			
Polyethylene	235.94	black	polynorbornene	319.82	yellow			

[continued]

Supplemental Table S3, continued.

Meconium			Placenta			Cord blood		
Types of microplastics	Size(um)	Colour	Types of microplastics	Size(um)	Colour	Types of microplastics	Size(um)	Colour
polyethylene	188.94	black	poly butene isotactic	184.33	yellow			
poly ethylene-co-acrylic acid	170.51	black	poly ethyl acrylate-co-methacrylic acid-co-3-(1-isocyanato-2-methyl ethyl)-	313.36	yellow			
polyethylene	168.66	black	polynorbornene	115.21	yellow			
poly propylene-co-1 -butene-co-ethylene	167.74	black	polynorbornene	145.62	yellow			
polyethylene	153.92	yellow	polynorbornene	160.37	yellow			
polyethylene	463.59	yellow	polyisoprene hydrogenated	116.13	yellow			
poly ethylene glycol ehtylether methacrylate	554.84	yellow	cellulose	270.05	black			
polycaprolactone	352.07	yellow	polynorbornene	118.89	yellow			
polyethylene	600.92	yellow	cellulose	250.69	yellow			
poly vinyl alcohol	178.80	black						
poly vinyl alcohol-co-ethylene	513.36	black						
polycaprolactone	337.33	black						
polycaprolactone	186.18	black						
polycaprolactone	296.77	black						
polyethylene	281.11	black						
polyethylene	364.06	black						
poly butadiene phenyl terminated	630.41	black						
poly N-vinylpyrrolidone-co-vinyl acetate	421.20	black						

[continued]

Supplemental Table S3, continued.

Meconium			Placenta			Cord blood		
Types of microplastics	Size(um)	Colour	Types of microplastics	Size(um)	Colour	Types of microplastics	Size(um)	Colour
polycaprolactone	574.19	black						
poly vinyl alcohol-co-ethylene	322.58	black						
polycaprolactone	183.41	black						
polyethylene	281.11	black						
polyethylene	194.47	black						
polyethylene	470.97	black						
poly ethylene-co-1-butene	151.15	black						
polyethylene	128.11	black						
polydimethylsiloxane-co-3-2-2-hydroxyethexy ethexy	107.83	transparent						
poly ethylene-co-vinyl acetate	203.69	transparent						
polyethylene	228.57	black						
polyepichlorohydrin	200.92	black						
cellulose	576.04	transparent						
polyethylene	104.15	black						
poly vinyl stearate	110.60	black						
poly vinyl stearate	129.03	black						
polyethylene	154.84	black						
polyethylene	138.25	black						
polyethylene	199.08	black						
polyethylene	129.03	black						
polyethylene	488.48	black						
polyethylene	338.25	black						
polyethylene	223.04	black						

[continued]

Supplemental Table S3, continued.

Meconium			Placenta			Cord blood		
Types of microplastics	Size(um)	Colour	Types of microplastics	Size(um)	Colour	Types of microplastics	Size(um)	Colour
polyethylene	153.92	black						
polyethylene	273.73	black						
polyamide resin	157.60	yellow						
poly ethylene terephthlate	81.11	black						
cellulose	331.80	black						
cellulose	257.14	black						
cellulose	296.77	black						
polyethylene	287.56	black						
polyethylene	336.41	black						
poly propylene-co-1-butene-co-ethylene	323.50	black						
poly ethylene-co-1-butene	281.11	black						
poly ethylene-co-2-butene	192.63	black						
poly propylene-co-1-butene-co-ethylene	136.41	black						
poly ethylene-co-1-butene	244.24	black						

Note: Merge similar types of plastics into MPs blend, which includes poly dimer acid-co-alkyl polyamine, poly ethyl acrylate-co-methacrylic acid-co-3-(1-isocyanato-2-methyl ethyl), poly ethyl acrylate-co-methacrylic acid-co-3-(1-isocyanato-1-methyl ethyl), poly ethylene-co methyl acrylate-co-glycidyl methacrylate, poly ethylene-co-1-butene, poly ethylene-co-2-butene, poly ethylene-co-acrylic acid, poly ethylene-co-ethyl acrylate, poly ethylene-co-octene, poly ethylene-co-vinyl acetate, poly lauryl mathacrylate-co-ethylene glycol dimethacrylate, poly N-vinylpyrrolidone-co-vinyl acetate, poly propylene-co-1-butene-co-ethylene, poly vinyl alcohol-co-ethylene and polydimethylsiloxane-co-3-2-2-hydroxyethexy ethexy.

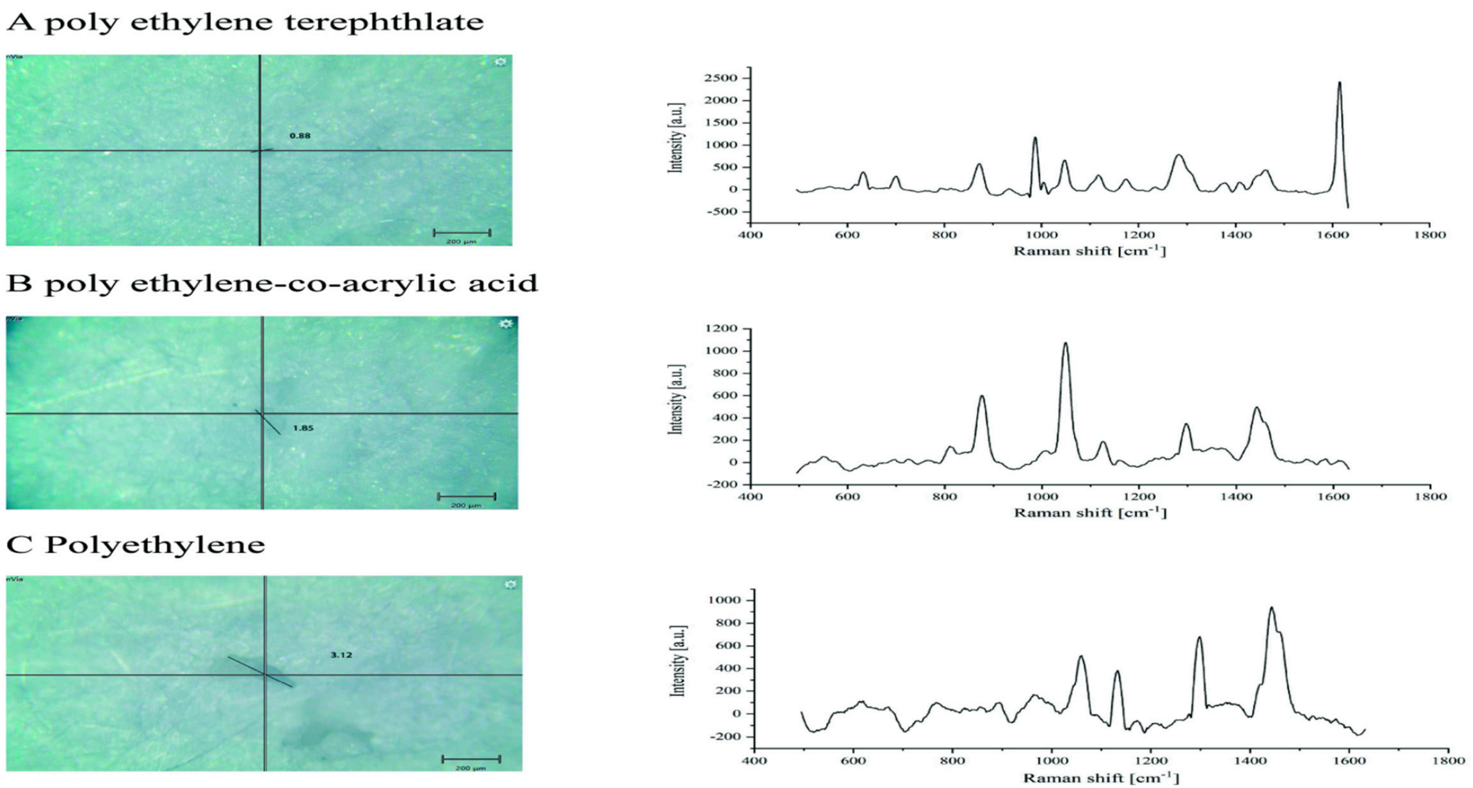

Figure S1: Experimental spectra with micro-photographs of the corresponding fragment places on the filter for (A) poly ethylene terephthlate from meconium of Num.8 , (B) poly ethylene-co-acrylic acid from sample meconium of Num.6, (C) Polyethylene from sample meconium of Num.7.

SPD/DS015-01-A May 2011
Data Sheet from the Spectroscopy Products Division

RENISHAW
apply innovation™

Renishaw Raman Database of Polymers

- 267 entries

Information stored with each entry:

- Systematic name
- Common abbreviation
- Molecular formula
- Composition and information on ingredients
- Sample supplier
- Physical properties of polymer sample
- Experimental parameters

Sample list:

1 1,2-Polybutadiene (7% cis 1,4; 93% vinyl 1,2)
2 Cellulose acetate [CA] (Acetyl content 39.8%)
3 Cellulose acetate butyrate
4 Cellulose acetate propionate (0.6 wt.% acetyl, 42.5 wt.% propionyl)
5 Cellulose acetate propionate (2.5 wt.% acetyl, 46 wt.% propionyl)
6 Cellulose propionate
7 Cellulose triacetate
8 Ertalyte PET-P (engineering thermoplastic)
9 Ethyl cellulose [EC] (Ethoxyl content 48%)
10 Ethyl cellulose [EC] (Ethoxyl content 49%)
11 Ethyl cellulose [EC] (Ethoxyl content 50%)
12 Halar ECTFE (engineering thermoplastic)
13 Hydex 4101 (PBT Polyester, engineering thermoplastic)
14 Hydroxybutyl methyl cellulose
15 Hydroxypropyl cellulose (Mw: Approx 1,000,000 g/mol)
16 Hydroxypropyl cellulose (Mw: Approx 100,000 g/mol)
17 Hydroxypropyl cellulose (Mw: Approx 60,000 g/mol)
18 Hydroxypropyl methyl cellulose
19 Kel-F PCTFE (engineering thermoplastic)
20 Kynar PVDF (engineering thermoplastic)
21 Methyl cellulose
22 Nylon 11, Poly(undecanolactam)
23 Nylon 12, Poly(lauryllactam)
24 Nylon 6(3)T, Poly(trimethyl hexamethylene terephthalamide)
25 Nylon 6, Polycaprolactam
26 Nylon 6/10, Poly(hexamethylene sebacamide)
27 Nylon 6/12, Poly(hexamethylene dodecanediamide)
28 Nylon 6/6, Poly(hexamethylene adipamide)
29 Nylon 6/66, Poly(hexamethylene adipamide-co-caprolactam)
30 Nylon 6/9, Poly(hexamethylene nonanediamide)
31 Poly(1,3-propylene adipate)
32 Poly(1,4-butylene adipate)
33 Poly(1,4-butylene terephthalate) [PBT]
34 Poly(1,4-cyclohexanedimethylene terephthalate-co-ethylene terephthalate)
35 Poly(1,4-phenylene sulfide)
36 Poly(1-butene), isotactic [PB] (Density: 0.910 kg/dm3, Mw: Approx 185,000)
37 Poly(1-butene), isotactic [PB] (Density: 0.910 kg/dm3, Mw: Approx 570,000)
38 Poly(1-butene), isotactic [PB] (Density: 0.915 kg/dm3)
39 Poly(2,6-dimethyl-1,4-phenylene oxide)
40 Poly(2,6-dimethyl-p-phenylene oxide)
41 Poly(2-ethyl-2-oxazoline)
42 Poly(2-hydroxyethyl methacrylate)
43 Poly(2-vinylnaphthalene)
44 Poly(2-vinylpyridine-co-styrene)
45 Poly(3-hydroxybutyric acid)
46 Poly(3-hydroxybutyric acid-co-3-hydroxyvaleric acid), natural origin
47 Poly(4,4-dipropoxy-2,2-diphenyl propane fumarate)
48 Poly(4-bromostyrene)
49 Poly(4-chlorostyrene)
50 Poly(4-ethylstyrene-co-divinylbenzene)
51 Poly(4-methyl-1-pentene) [PMP]
52 Poly(4-methyl-1-pentene) [PMP] (High molecular weight, crystalline, isotactic)
53 Poly(4-methyl-1-pentene) [PMP] (Low molecular weight, low density thermoplast)
54 Poly(4-methyl-1-pentene) [PMP] (Medium molecular weight, isotactic)

1

Figure S2: Potential Microplastic Particle Species Identification Certificate, First Page.

Renishaw plc
Spectroscopy Products Division
Old Town, Wotton-under-Edge,
Gloucestershire GL12 7DW
United Kingdom

T: +44 1453 524524
F: +44 1453 523901
E: raman@renishaw.com
www.renishaw.com

RENISHAW apply innovation™

55 Poly(4-tert-butylstyrene)
56 Poly(4-vinylbiphenyl)
57 Poly(4-vinylpyridine)
58 Poly(4-vinylpyridine-co-styrene)
59 Poly(9-vinylcarbazole) (Average Mw: 1,100,000 g/mol)
60 Poly(9-vinylcarbazole) (Average Mw: 63,000 g/mol)
61 Poly(acrylamide-co-acrylic acid)
62 Poly(acrylic acid) (99.7%)
63 Poly(acrylic acid), 100%
64 Poly(acrylonitrile-co-butadiene-co-styrene) (45% butadiene)
65 Poly(acrylonitrile-co-butadiene-co-styrene) (high butadiene content)
66 Poly(alpha-methylstyrene) [PMS] (Mn: Approx 4,000 g/mol)
67 Poly(alpha-methylstyrene) [PMS] (Mn: Approx 790 g/mol)
68 Poly(alpha-methylstyrene) [PMS] (Mw: Approx 11,500 g/mol)
69 Poly(benzyl methacrylate)
70 Poly(Bisphenol A carbonate), Polycarbonate [PC]
71 Poly(Bisphenol A-co-epichlorohydrin), Phenoxy resin
72 Poly(butadiene) [PB] (1% 1,2-addition, 75% cis-1,4, 24% trans-1,4)
73 Poly(butadiene) [PB], (98% cis)
74 Poly(butadiene), phenyl terminated [PB] (Mn: Approx 1,000 g/mol)
75 Poly(butadiene), phenyl terminated [PB] (Mn: Approx 1,800 g/mol)
76 Poly(butyl acrylate) [PBA]
77 Poly(butyl methacrylate)
78 Poly(butyl methacrylate-co-isobutyl methacrylate) (50/50 copolymer)
79 Poly(butyl methacrylate-co-methyl methacrylate)
80 Poly(chlorotrifluoroethylene) [PCTFE] (engineering thermoplastic)
81 Poly(cyclohexyl methacrylate)
82 Poly(diallyl isophthalate)
83 Poly(diallyl phthalate) [PDAP]
84 Poly(dimer acid-co-alkyl polyamine) (Softening point: 140°C)
85 Poly(dimer acid-co-alkyl polyamine) (Softening point: 160°C)
86 Poly(dimethylsiloxane) (liquid, analysed through quartz cuvette)
87 Poly(dimethylsiloxane) ethoxylate/propoxylate
88 Poly(dimethylsiloxane-co-alkylmethylsiloxane)
89 Poly(dimethylsiloxane-co-diphenylsiloxane), dihydroxy terminated
90 Poly(dimethylsiloxane-co-methylphenylsiloxane), 510 fluid
91 Poly(ethyl methacrylate) (Mw: Approx 280,000 g/mol)
92 Poly(ethyl methacrylate) (Mw: Approx 515,000 g/mol)
93 Poly(ethyl methacrylate) (Mw: Approx 850,000 g/mol)
94 Poly(ethyl methacrylate-co-methyl acrylate)
95 Poly(ethylene adipate)
96 Poly(ethylene glycol) (Mn: Approx 10,000 g/mol)
97 Poly(ethylene glycol) (Mn: Approx 2,000 g/mol)
98 Poly(ethylene glycol) (Mn: Approx 4,600 g/mol)
99 Poly(ethylene glycol) (Mn: Approx 400 g/mol)
100 Poly(ethylene glycol) dimethyl ether
101 Poly(ethylene glycol) distearate
102 Poly(ethylene glycol) ethyl ether methacrylate
103 Poly(ethylene glycol) methacrylate
104 Poly(ethylene glycol) methyl ether (Mn: Approx 350 g/mol)
105 Poly(ethylene glycol) methyl ether (Mn: Approx 5,000 g/mol)
106 Poly(ethylene glycol) monooleate
107 Poly(ethylene glycol), reacted with Bisphenol A diglycidyl ether
108 Poly(ethylene glycol)-block-poly(propylene glycol)-block-poly(ethylene glycol)
109 Poly(ethylene glycol-ran-propylene glycol)
110 Poly(ethylene oxide) [PEOX] (>95% Poly(ethylene oxide))
111 Poly(ethylene oxide) [PEOX] (100% PEOX, Mv: Approx 200,000 g/mol)
112 Poly(ethylene oxide) [PEOX] (100% PEOX, Mv: Approx 600,000 g/mol)
113 Poly(ethylene terephthalate) [PET]
114 Poly(ethylene-co-1-butene) [E/B]
115 Poly(ethylene-co-1-butene-co-1-hexene)
116 Poly(ethylene-co-1-octene)
117 Poly(ethylene-co-acrylic acid)
118 Poly(ethylene-co-ethyl acrylate) [E/EA]
119 Poly(ethylene-co-methacrylic acid), sodium salt [E/MA]
120 Poly(ethylene-co-methyl acrylate-co-acrylic acid)
121 Poly(ethylene-co-methyl acrylate-co-glycidyl methacrylate)
122 Poly(ethylene-co-propylene) [E/P]
123 Poly(ethylene-co-tetrafluoroethylene) [E/TFE]
124 Poly(ethylene-co-vinyl acetate) [E/VAC] (60/40 copolymer, 40% vinyl acetate)
125 Poly(ethylene-co-vinyl acetate) [E/VAC] (67/33 copolymer, 33% vinyl acetate)
126 Poly(ethylene-co-vinyl acetate) [E/VAC] (72/28 copolymer, 28% vinyl acetate)

2 of 4

2

Figure S3: Potential Microplastic Particle Species Identification Certificate, Second Page.

2

Renishaw plc
Spectroscopy Products Division
Old Town, Wotton-under-Edge,
Gloucestershire GL12 7DW
United Kingdom

T: +44 1453 524524
F: +44 1453 523901
E: raman@renishaw.com
www.renishaw.com

RENISHAW apply innovation™

127 Poly(ethylene-co-vinyl acetate) [E/VAC] (75/25 copolymer, 25% vinyl acetate)
128 Poly(ethylene-co-vinyl acetate) [E/VAC] (82/18 copolymer, 82% ethylene)
129 Poly(ethylene-co-vinyl acetate) [E/VAC] (86/14 copolymer, 86% ethylene)
130 Poly(isobornyl methacrylate)
131 Poly(isobutyl methacrylate) (Mw: Approx 260,000 g/mol)
132 Poly(isobutyl methacrylate) (Mw: Approx 70,000 g/mol)
133 Poly(isobutylene) [PIB] (Mw: Approx 4,200,000 g/mol)
134 Poly(isobutylene) [PIB] (Mw: Approx 500,000 g/mol)
135 Poly(isopropyl methacrylate)
136 Poly(lauryl methacrylate-co-ethylene glycol dimethacrylate)
137 Poly(methyl methacrylate) [PMMA] (Mw: Approx 15,000 g/mol)
138 Poly(methyl methacrylate) [PMMA] (Mw: Approx 35,000 g/mol)
139 Poly(methyl methacrylate) [PMMA] (Mw: Approx 996,000 g/mol)
140 Poly(methyl methacrylate), isotactic [PMMA] (>80% isotactic)
141 Poly(methyl methacrylate-co-butyl methacrylate)
142 Poly(methyl methacrylate-co-ethylene glycol dimethacrylate)
143 Poly(methyl vinyl ether)
144 Poly(methyl vinyl ether-alt-maleic acid)
145 Poly(methyl vinyl ether-alt-maleic anhydride)
146 Poly(methylphenylsiloxane), 710 fluid
147 Poly(N,N'-(1,3-phenylene) isophthalamide)
148 Poly(n-butyl methacrylate)
149 Poly(neopentyl glycol adipate)
150 Poly(N-vinylpyrrolidone-co-vinyl acetate)
151 Poly(oxymethylene), Polyformaldehyde [POM] (engineering thermoplastic) (Melt index: 16g/10min)
152 Poly(oxymethylene), Polyformaldehyde [POM] (engineering thermoplastic) (Melt index: 6g/10min)
153 Poly(phenylene sulfide)
154 Poly(p-phenylene ether-sulfone)
155 Poly(propylene glycol) dimethacrylate
156 Poly(propylene glycol), poly(propylene oxide) [PPOX] (Mn: Approx 3,500 g/mol)
157 Poly(propylene glycol), poly(propylene oxide) [PPOX] (Mn: Approx 425 g/mol)
158 Poly(propylene glycol), poly(propylene oxide) [PPOX] (Mn: Approx 725 g/mol)
159 Poly(propylene-co-1-butene) [P/B] (Crystal. random copolymer, 14 wt.% 1-butene)
160 Poly(propylene-co-1-butene) [P/B] (Crystal. random copolymer, 8 wt.% 1-butene)
161 Poly(propylene-co-1-butene-co-ethylene) [P/B/E]
162 Poly(propylene-co-ethylene) [E/P] (Amorphous copolymer)
163 Poly(propylene-co-ethylene) [E/P] (isotactic random, cont. 5-15% ethylene)
164 Poly(sodium 4-styrenesulfonate)
165 Poly(styrene-alt-maleic anhydride)
166 Poly(styrene-co-acrylonitrile) [S/A] [AS] (25% Acrylonitrile)
167 Poly(styrene-co-acrylonitrile) [S/A] [AS] (32% Acrylonitrile)
168 Poly(styrene-co-allyl alcohol) [S/AA]
169 Poly(styrene-co-butadiene) [S/B] (30% Styrene, ABA block copolymer)
170 Poly(styrene-co-butadiene) [S/B] (45 wt.% styrene)
171 Poly(styrene-co-butyl methacrylate)
172 Poly(styrene-co-isoprene)
173 Poly(tert-butyl methacrylate)
174 Poly(tetrafluoroethylene) [PTFE]
175 Poly(vinyl acetate) [PVAC] (>98% Poly(vinyl acetate), Mw: Approx 260,000 g/m)
176 Poly(vinyl acetate) [PVAC] (100% Poly(vinyl acetate), Mw: Approx 12,800 g/mol)
177 Poly(vinyl acetate) [PVAC] (100% Poly(vinyl acetate), Mw: Approx 500,000 g/mol)
178 Poly(vinyl alcohol) [PVAL] (80% hydrolyzed)
179 Poly(vinyl alcohol) [PVAL] (87-89% hydrolyzed)
180 Poly(vinyl alcohol) [PVAL] (98% hydrolyzed)
181 Poly(vinyl alcohol) [PVAL] (99.7% hydrolyzed)
182 Poly(vinyl alcohol) [PVAL] (99+% hydrolyzed)
183 Poly(vinyl alcohol-co-ethylene) [VAL/E]
184 Poly(vinyl alcohol-co-vinyl butyral)
185 Poly(vinyl butyral-co-vinyl alcohol-co-vinyl acetate) [VB/VAL/VAC]
186 Poly(vinyl chloride) [PVC] (Mw: Approx 233,000 g/mol)
187 Poly(vinyl chloride) [PVC] (Mw: Approx 275,000 g/mol)
188 Poly(vinyl chloride) [PVC] (Mw: Approx 80,000 g/mol)
189 Poly(vinyl chloride), carboxylated [PVC-C] (1.8% carboxyl)
190 Poly(vinyl chloride-co-vinyl acetate) (88/12 copolymer)
191 Poly(vinyl chloride-co-vinyl acetate) (90/10 copolymer)
192 Poly(vinyl chloride-co-vinyl acetate), carboxylated (86/13 copolymer)
193 Poly(vinyl chloride-co-vinyl acetate-co-2-hydroxypropyl acrylate)
194 Poly(vinyl chloride-co-vinyl acetate-co-vinyl alcohol)
195 Poly(vinyl cinnamate)
196 Poly(vinyl formal)
197 Poly(vinyl methyl ketone)
198 Poly(vinyl stearate)

3 of 4

3

Figure S4: Potential Microplastic Particle Species Identification Certificate, Third Page.

Renishaw plc
Spectroscopy Products Division
Old Town, Wotton-under-Edge,
Gloucestershire GL12 7DW
United Kingdom

T: +44 1453 524524
F: +44 1453 523901
E: raman@renishaw.com
www.renishaw.com

RENISHAW
apply innovation™

199 Poly(vinylidene chloride-co-acrylonitrile)
200 Poly(vinylidene chloride-co-vinyl chloride)
201 Poly(vinylidene flouride) [PVDF] (Mw: Approx 180,000 g/mol)
202 Poly(vinylidene flouride) [PVDF] (Mw: Approx 534,000 g/mol)
203 Poly(vinylidene fluoride) [PVDF] (59% Fluorine, Mw: Approx 530,000 g/mol)
204 Poly[4,4'-methylenebis(phenyl isocyanate)-alt-1,4-butanediol/poly(butylene adipate)]
205 Poly[dimethylsiloxane-co-[3-[2-(2-hydroxyethoxy)ethoxy]propyl]methylsiloxane]
206 Poly[ethyl acrylate-co-methacrylic acid-co-3-(1-isocyanato-1-methyl ethyl)-alpha-methylstyrene]
207 Poly[methyl(3,3,3-trifluoropropyl)siloxane]
208 Polyacenaphthylene
209 Polyacrylamide carboxyl modified
210 Polyacrylamide, non-ionic
211 Polyamide resin
212 Polybutadiene-block-polyisoprene
213 Polycaprolactone (Mn: Approx 80,000 g/mol)
214 Polycaprolactone (Mn: Approx 9,300 g/mol)
215 Polycaprolactone diol (Mn: Approx 1,250 g/mol)
216 Polycaprolactone diol (Mn: Approx 2,000 g/mol)
217 Polycaprolactone triol (Mn: Approx 300 g/mol)
218 Polycaprolactone triol (Mn: Approx 900 g/mol)
219 Polyepichlorohydrin
220 Polyethylene [PE] (Specific gravity: 0.906, Mw: Approx 35,000 g/mol)
221 Polyethylene [PE] (Specific gravity: 0.920, Mw: Approx 4,000 g/mol)
222 Polyethylene [PE] (Specific gravity: 0.930, Mw: Approx 15,000 g/mol)
223 Polyethylene, chlorinated [PE-C] (25% chlorinated)
224 Polyethylene, chlorinated [PE-C] (36% chlorinated)
225 Polyethylene, chlorinated [PE-C] (42% chlorinated)
226 Polyethylene, chlorinated [PE-C] (48% chlorinated)
227 Polyethylene, chlorosulfonated [PE-CS]
228 Polyethylene, high density [PE-HD] (Density: 0.95 kg/dm3)
229 Polyethylene, linear low density [PE-LLD] (Density: 0.918 kg/dm3)
230 Polyethylene, low density [PE-LD] (Density: 0.923 kg/dm3)
231 Polyethylene, medium density [PE] (Density: 0.940 kg/dm3)
232 Polyethylene, oxidized
233 Polyethylene, spectrophotometric grade [PE]
234 Polyethylene, ultra high molecular weight [PE] (Mw: 3,000,000-6,000,000 g/mol)
235 Polyethylene-graft-maleic anhydride
236 Polyisoprene, chlorinated
237 Polyisoprene, hydrogenated
238 Polyisoprene, trans
239 Polynorbornene
240 Polyoxyethylene(18) tridecyl ether
241 Polyoxymethylene, Poly(trioxane), Polyformaldehyde, Polyacetal [POM]
242 Polyphenylsulfone
243 Polypropylene, chlorinated [PP-C] (isotactic, 26% chlorine)
244 Polypropylene, chlorinated [PP-C] (isotactic, 26% wt. chlorine)
245 Polypropylene, isotactic [PP] (Density: 0.90 kg/dm3)
246 Polypropylene, isotactic [PP] (Density: 0.902 kg/dm3)
247 Polypropylene, syndiotactic [PP] (93% syndiotactic, Density: 0.900 kg/dm3)
248 Polystyrene [PS] (Mw: Approx 280,000 g/mol)
249 Polystyrene standard [PS] (Mw: Approx 2,000,000 g/mol)
250 Polystyrene standard [PS] (Mw: Approx 50,000 g/mol)
251 Polystyrene standard [PS] (Mw: Approx 600,000 g/mol)
252 Polystyrene standard [PS] (Mw: Approx 800 g/mol)
253 Polystyrene, isotactic [PS] (90% isotactic, Mw: Approx 400,000 g/mol)
254 Polystyrene-co-poly(ethylene-ran-butylene)
255 Polytetrahydrofuran, alpha-hydro-omega-hydroxy-poly(oxy-1,4-butanediyl)
256 Polyvinylpyrrolidone [PVP] (Mw: Approx 1,300,000 g/mol)
257 Polyvinylpyrrolidone [PVP] (Mw: Approx 10,000 g/mol)
258 Polyvinylpyrrolidone [PVP] (Mw: Approx 360,000 g/mol)
259 Polyvinyltoluene
260 Pomalux SD-A (engineering thermoplastic)
261 Semitron ESd 225 (engineering thermoplastic)
262 Techtron PPS (engineering thermoplastic)
263 Teflon FEP (engineering thermoplastic)
264 Teflon PFA (engineering thermoplastic)
265 Teflon PTFE (engineering thermoplastic)
266 Tefzel ETFE (engineering thermoplastic)
267 Ultem 1000 PEI (engineering thermoplastic)

4 of 4

Figure S5: Potential Microplastic Particle Species Identification Certificate, Fourth Page.
